# Comparison of azithromycin and doxycycline for the treatment of *Chlamydia psittaci* pneumonia

**DOI:** 10.3389/fphar.2026.1900992

**Published:** 2026-10-16

**Authors:** Ran Cheng, Zhonghua Deng, Fei Lin, Biying Zhang, Jingjin Liang, Ming Lu

**Affiliations:** 1 Department of Infectious Diseases, Peking University Third Hospital, Beijing, China; 2 Department of Respiratory and Critical Care Medicine, Peking University Third Hospital, Beijing, China

**Keywords:** azithromycin, *Chlamydia* *psittaci* pneumonia, doxycycline, next-generation sequencing, prognosis

## Abstract

**Background and Objective:**

*Chlamydia psittaci* pneumonia (CPP) has received increasing clinical attention worldwide in recent years. Although doxycycline is the recommended first-line therapy and macrolides serve as alternatives, no studies have directly compared their clinical efficacy in the general adult population. This study aimed to compare clinical outcomes between doxycycline and azithromycin in adult patients with CPP.

**Methods:**

This retrospective single-center cohort study included 57 adult patients with CPP at Peking University Third Hospital from 2021 to 2025, of whom 31 received doxycycline and 26 received azithromycin. Demographic characteristics, laboratory findings, and clinical outcomes were compared between the two groups.

**Results:**

Baseline characteristics, including comorbidities and laboratory findings, were comparable between groups. Respiratory support requirements and glucocorticoid use were similar (all *P* > 0.05). A numerical imbalance in Intensive Care Unit (ICU) admission rate was observed, with 16.1% in the doxycycline group and 0% in the azithromycin group (*P* = 0.056); this difference likely reflects treatment-selection bias given the small sample size. No significant differences were observed in 28-day discharge rate (96.8% vs. 100%), mortality (3.2% vs. 0%), median length of hospital stay (6 vs. 6 days, *P* = 0.691), or time to defervescence (2 vs. 2 days, *P* = 0.436).

**Conclusion:**

No statistically significant differences were observed in measured short-term clinical outcomes between doxycycline and azithromycin for CPP. Both may be reasonable therapeutic options, but findings should be interpreted cautiously given limited statistical power and potential confounding by indication, especially where drug availability or susceptibility data are limited.

## 1 Introduction

Psittacosis, a zoonotic infectious disease, is caused by *Chlamydia psittaci* (*C. psittaci*), an obligate intracellular bacterium (Chu et al., 2026). Previous studies have reported that *C. psittaci* accounted for 1.03% of all cases of community-acquired pneumonia (CAP) (Hogerwerf et al., 2017). In another study focusing on adults with severe CAP investigated using intensive molecular diagnostic methods, the detection rate of *C. psittaci* was reported to be as high as 8% (Wu et al., 2020). The clinical presentation of *Chlamydia psittaci* pneumonia (CPP) is highly heterogeneous, encompassing a broad spectrum of disease severity (Homma et al., 2011; Shapiro et al., 1992). Clinical manifestations range from mild influenza-like symptoms to rapidly progressive, life-threatening disease (Beeckman and Vanrompay, 2009). Given this potential for rapid clinical deterioration, prompt and appropriate antimicrobial therapy is essential to prevent disease progression and improve patient outcomes (Huang et al., 2025; Song et al., 2025). The selection of optimal therapeutic agents is guided by *in vitro* susceptibility data, clinical experience, and illness severity (Chu et al., 2026). Currently, three major classes of antibiotics have activity against *C. psittaci*: tetracyclines, macrolides, and fluoroquinolones.

Tetracyclines, particularly doxycycline, remain the cornerstone of CPP treatment and are universally recommended as first-line agents (Chu et al., 2026). Doxycycline exhibits excellent intracellular penetration and potent bacteriostatic activity against *C. psittaci* by reversibly binding to the 30S ribosomal subunit, thereby inhibiting bacterial protein synthesis and arresting intracellular replication. Older-generation tetracyclines (e.g., tetracycline, oxytetracycline) are well established to carry risks of permanent dental discoloration and impaired bone development, and are contraindicated in pregnant women and young children. In contrast, accumulating evidence indicate that short-course doxycycline is associated with minimal risk of dental staining, and may be used across all pediatric age groups when clinically indicated (Ma et al., 2025; Wormser et al., 2019). Nevertheless, regulatory labeling and clinical practice recommendations for doxycycline in young children vary across regions. Macrolides therefore remain clinically useful alternatives for children, pregnant women, and other patients for whom doxycycline is unsuitable (Tetracycline MotherToBaby Fact Sheets, 1994).

Macrolides, notably azithromycin and clarithromycin, serve as important alternatives for patients with contraindications or intolerance to tetracyclines. Azithromycin offers favorable pharmacokinetic properties, including excellent tissue and intracellular penetration, a prolonged half-life allowing once-daily dosing, and good gastrointestinal tolerability (Sandman and Iqbal, 2024). Mechanistically, azithromycin binds to the 50S ribosomal subunit, suppressing bacterial protein synthesis through a distinct yet functionally convergent pathway. Macrolides are particularly valuable for treating pediatric patients and pregnant women, providing safe and effective therapeutic alternatives to tetracyclines.

Early retrospective evidence suggested that erythromycin was at least as effective as tetracycline for treating *C. psittaci* pneumonia in a small cohort of patients with acute lower respiratory tract infection (Hammers-Berggren et al., 1991). However, these findings were limited by the small sample size, the use of first-generation macrolides rather than modern agents such as azithromycin, and the absence of comprehensive pharmacological characterization. To date, no robust comparative studies investigating the therapeutic efficacy of azithromycin-based versus doxycycline-based regimens have been reported for the population with confirmed CPP. Accordingly, we performed a retrospective comparative analysis of clinical outcomes between patients treated with azithromycin and doxycycline for confirmed CPP.

## 2 Methods

### 2.1 Study design

We conducted a retrospective, single-center cohort analysis of adult patients diagnosed with CPP at Peking University Third Hospital from 1 January 2021, to 31 December 2025. Eligible participants were required to meet the following inclusion criteria: (1) age ≥18 years; (2) fulfillment of the diagnostic criteria for CAP according to established guidelines; (3) confirmation of *C. psittaci* infection through metagenomic next-generation sequencing (mNGS) or targeted next-generation sequencing (tNGS) performed on bronchoalveolar lavage fluid, serum, or sputum specimens; and (4) treatment with either doxycycline 100 mg twice daily or azithromycin 500 mg once daily as the sole antimicrobial therapy. Patients were excluded if they: (1) lacked molecular diagnostic confirmation via NGS-based methods; (2) had incomplete or unavailable medical records; or (3) received any antimicrobial regimens other than doxycycline or azithromycin (including alternative monotherapy or combination therapies).

### 2.2 Ethical considerations

The study protocol conformed to the ethical principles of the Declaration of Helsinki and was approved by the Institutional Review Board of Peking University Third Hospital, Beijing, China (approval No. 20250641). The requirement for written informed consent was waived due to the anonymized retrospective nature of the analysis. The data were analyzed anonymously.

### 2.3 Statistical analysis

Continuous variables with a normal distribution were expressed as mean ± standard deviation and compared using the independent-samples t-test, while non-normally distributed continuous variables were presented as median (interquartile range) and compared using the Mann-Whitney U test. Categorical variables were expressed as counts (percentages) and compared using the chi-square test or Fisher’s exact test, as appropriate. All statistical analyses were performed using SPSS software (version 22.0; IBM Corp., Armonk, NY, United States). A two-tailed *P* value <0.05 was considered statistically significant.

### 2.4 NGS analysis

The specimens were sent to the Department of Clinical Laboratory of Peking University Third Hospital for pathogenic mNGS (PACEseq, Hugobiotech, Beijing, China) and tNGS (DRseq, Hugobiotech, Beijing, China) detection.

### 2.5 Data collection and clinical definitions

Comprehensive clinical data were retrospectively extracted from electronic medical records by two independent investigators using a standardized data collection form, with discrepancies resolved through consensus discussion with a third senior investigator. Patient characteristics, including age, sex, smoking history, comorbidities, and avian contact history, were documented alongside clinical manifestations, vital signs, and laboratory findings. Computed tomography characteristics were evaluated for the location of lung consolidation, the presence of pleural effusion. Antimicrobial regimens and clinical outcomes were also assessed, encompassing 28-day all-cause mortality, intensive care unit admission, time to defervescence (defined as maintenance of body temperature <37.3 °C for ≥48 h without antipyretic administration), length of hospital stay (calculated from admission to discharge or death), and 28-day discharge rate.

### 2.6 Antimicrobial treatment regimens

Patients in the azithromycin group received intravenous azithromycin at a dosage of 500 mg once daily. Those in the doxycycline group received intravenous doxycycline at a dosage of 100 mg twice daily. For patients demonstrating clinical improvement, a step-down transition from intravenous to oral administration was implemented. Clinical improvement was defined as defervescence (body temperature <37.3 °C for ≥48 h without antipyretics), resolution of respiratory symptoms, and improvement in inflammatory markers (decline in C-reactive protein and procalcitonin levels). Oral azithromycin was administered at 500 mg once daily, whereas oral doxycycline was continued at 100 mg twice daily. The total duration of antimicrobial therapy ranged from 10 to 14 days for both groups, with the exact duration determined at the discretion of the attending physician based on clinical response and laboratory parameters. For patients with severe illness, delayed clinical response, or persistent radiological infiltrates, the treatment course was extended up to 21 days.

## 3 Results

### 3.1 Comparison of demographic and clinical characteristics

A total of 57 patients were included in the analysis, with 31 receiving doxycycline and 26 receiving azithromycin (Figure 1). The demographic and clinical characteristics were comparable between the two groups (Table 1). The median age was 61 years in the doxycycline group and 63 years in the azithromycin group (*P* = 0.911). The proportion of patients aged ≥65 years was similar between groups (41.9% vs. 42.3%, *P* = 0.977). Most patients were male in both groups (45.2% vs. 38.5%, *P* = 0.610), and no significant differences were observed in smoking, drinking, or history of poultry exposure (all *P* > 0.05).

**FIGURE 1:**
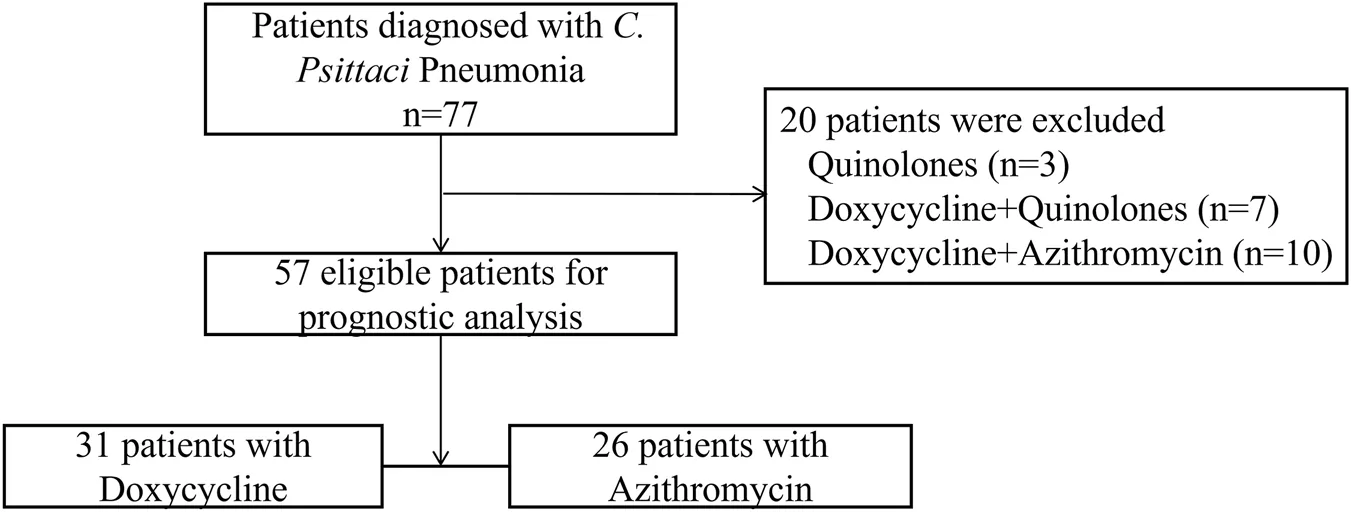
Flowchart of the study.

**TABLE 1:** Comparison of demographic and clinical characteristics between patients with doxycycline and azithromycin.

Variable	Doxycycline (n = 31)	Azithromycin (n = 26)	*P*-value
Age, median (IQR), years	61 (22)	63 (21.75)	0.911*
Time from illness onset to hospital admission	5 (3)	5 (4.75)	0.440*
Age ≥65 years, n (%)	13 (41.9)	11 (42.3)	0.977^†^
Male, n (%)	14 (45.2)	10 (38.5)	0.610^†^
Smoking, n (%)	8 (25.8)	5 (19.2)	0.556^†^
Drinking, n (%)	8 (25.8)	7 (26.9)	0.924^†^
History of poultry exposure, n (%)	21 (67.7)	20 (76.9)	0.442^†^
Comorbidities, n (%)			
Hypertension	11 (35.5)	8 (26.0)	0.707^†^
Diabetes	5 (16.1)	2 (7.7)	0.436^†^
Heart disease	3 (9.7)	2 (7.7)	1.000^†^
Immunosuppressive disease	2 (6.5)	2 (7.7)	1.000^†^
Chronic lung disease	2 (6.5)	0	0.495^†^
Chronic kidney diseases	2 (6.5)	0	0.495^†^
Cerebrovascular diseases	3 (9.7)	2 (7.7)	1.000^†^
Malignancy	3 (9.7)	1 (3.8)	0.617^†^
Symptom, n (%)			
Fever	31 (100)	26 (100)	-
Cough	18 (58.1)	13 (50)	0.543^†^
Sputum	10 (32.3)	4 (15.4)	0.217^†^
Dyspnea	6 (19.4)	4 (15.4)	0.741^†^
Headache	15 (48.4)	12 (46.2)	0.866^†^
Dizziness	9 (29.0)	11 (42.3)	0.296^†^
Delirium	3 (9.7)	5 (19.2)	0.448^†^
Vomit/Nausea	7 (22.6)	6 (23.1)	0.965^†^
Diarrhea/abdominal pain	4 (12.9)	2 (7.7)	0.678^†^
Urinary system symptoms	2 (6.5)	0	0.495^†^

* This P value was calculated with the use of Mann-Whitney U test.

^†^ This P value was calculated with the use of chi-square test or Fisher’s exact test.

Regarding comorbidities, the prevalence of hypertension, diabetes, heart disease, immunosuppressive disease, chronic lung disease, chronic kidney disease, cerebrovascular disease, and malignancy was comparable between groups (all *P* > 0.05). Clinically, fever was a universal symptom in both groups (100% in each). Other common symptoms, including cough, sputum production, dyspnea, headache, and gastrointestinal symptoms, did not differ significantly between the two groups (all *P* > 0.05).

### 3.2 Laboratory and imaging findings at admission

Laboratory parameters at admission were similar between the doxycycline and azithromycin groups (all *P* > 0.05) (Table 2). For routine blood tests, the median white blood cell count, neutrophil count, lymphocyte count, hemoglobin, and platelet count showed no significant differences between the two groups (all *P* > 0.05). Biochemical tests showed that alanine aminotransferase (ALT) and aspartate aminotransferase (AST) levels were elevated in approximately half of the patients. However, between-group comparative analysis revealed no significant differences in AST, ALT, lactate dehydrogenase (LDH), total bilirubin, albumin, serum sodium, creatinine, or creatine kinase between the two groups (all *P* > 0.05). For infection biomarkers, C-reactive protein (CRP) was elevated in 90% of patients. Levels of inflammatory markers, including CRP and procalcitonin, were not significantly different (all *P* > 0.05). Other parameters, including arterial partial pressure of oxygen, body temperature, and D-dimer levels, were also similar between groups (all *P* > 0.05).

**TABLE 2:** Laboratory characteristics at admission in patients with doxycycline and azithromycin.

Variable	Doxycycline (n = 31)	Azithromycin (n = 26)	*P*-value
Blood routine, median (IQR)			
White blood cell count, ×10^9^/L	7.51 (3.24)	6.51 (3.83)	0.206*
Neutrophil count, ×10^9^/L	5.81 (3.16)	5.48 (3.16)	0.259*
Lymphocyte count, ×10^9^/L	0.81 (0.63)	0.81 (0.71)	0.631*
Hemoglobin, g/L	131 (17)	129 (26.75)	0.968*
Platelet count, ×10^9^/L	199 (95)	187.5 (68.75)	0.804*
Blood biochemistry, median (IQR)			
Aspartate aminotransferase, U/L	43.7 (53.5)	35.3 (42.63)	0.689*
Alanine aminotransferase, U/L	51 (58.5)	47.05 (64.25)	0.677*
Lactate dehydrogenase, U/L	278 (190.75)	283.5 (175.25)	0.828*
Total bilirubin, μmol/L	12.9 (7.3)	15.3 (12.03)	0.136*
Albumin, g/L	37.6 (10.4)	37.5 (8.4)	0.397*
Serum sodium, mmol/L	134.5 (6.5)	134.75 (6.78)	0.773*
Creatinine, μmol/L	83 (51)	71 (23.75)	0.294*
Creatine kinase, U/L	102.5 (460.5)	85 (275.5)	0.589*
Inflammatory mediators, median (IQR)			
C-reactive protein, mg/L	170.3 (145.3)	146.9 (79.95)	0.352*
Bacterial infection mediators, median (IQR)			
Procalcitonin, μg/L	0.4 (2.18)	0.27 (0.60)	0.283*
Vital signs, median (IQR)			
PaO2, mmHg	71 (14.27)	70.45 (15.95)	0.864*
Temperature, ◦C	39.1 (1.3)	39.15 (1.35)	0.878*
Blood coagulation, median (IQR)			
D-dimer, μg/mL	0.42 (1.13)	0.38 (0.64)	0.520*
Radiographic characteristics, n (%)			
Lesions in isolated left lung	12 (38.7)	13 (50)	0.392^†^
Lesions in isolated right lung	11 (35.5)	6 (23.1)	0.308^†^
Lesions in bilateral lung	8 (25.8)	7 (26.9)	0.924^†^
Image changes computed tomography scans, n (%)			
Ground-glass opacity	15 (48.4)	11 (42.3)	0.646^†^
Consolidation	25 (80.6)	23 (88.5)	0.488^†^
Pleural effusion	7 (22.6)	6 (23.1)	0.965^†^

* This P value was calculated with the use of Mann-Whitney U test.

^†^ This P value was calculated with the use of chi-square test or Fisher’s exact test.

High-resolution thoracic CT showed consolidation as the dominant pattern in both groups, followed by ground-glass opacities (GGO) and pleural effusions (Table 2). There were no statistically significant differences between the groups in anatomical distribution or in the prevalence of consolidation and pleural effusion (all *P* > 0.05).

### 3.3 Treatment and clinical outcomes

Regarding respiratory support, 29.0% of patients in the doxycycline group and 38.5% in the azithromycin group required no respiratory support (*P* = 0.452) (Table 3). Conventional oxygen therapy was the most common respiratory support modality, used in 54.8% and 53.8% of patients, respectively (*P* = 0.940). High-flow nasal cannula and non-invasive mechanical ventilation were used in a small number of patients with comparable frequencies between groups. Invasive mechanical ventilation was required in 6.5% of patients in the doxycycline group but none in the azithromycin group (*P* = 0.495).

**TABLE 3:** Treatments and prognosis of patients with doxycycline and azithromycin.

Variable	Doxycycline (n = 31)	Azithromycin (n = 26)	*P*-value
Respiratory support mode, n (%)			
No respiratory support	9 (29.0)	10 (38.5)	0.452^†^
Conventional oxygen therapy	17 (54.8)	14 (53.8)	0.940^†^
High-flow nasal cannula	1 (3.2)	1 (3.8)	1.000^†^
Non-invasive mechanical ventilation	2 (6.5)	1 (3.8)	1.000^†^
Invasive mechanical ventilation	2 (6.5)	0	0.495^†^
Other supportive treatments, n (%)			
Glucocorticoids	4 (12.9)	5 (19.2)	0.718^†^
Continuous renal replacement therapy	2 (6.5)	0	0.495^†^
Clinical outcomes			
Intensive care unit admission, n (%)	5 (16.1)	0	0.056^†^
Hospital discharge within 28 days, n (%)	30 (96.8)	26 (100)	1.000^†^
Hospital death, n (%)	1 (3.2)	0	1.000^†^
Hospital stay, median (IQR)	6 (4)	6 (3)	0.691*
Defervescence time, median (IQR)	2 (2)	2 (1)	0.436*

* This P value was calculated with the use of Mann-Whitney U test.

^†^ This P value was calculated with the use of chi-square test or Fisher’s exact test.

Intensive care unit (ICU) admission occurred in 16.1% of patients in the doxycycline group compared to none in the azithromycin group (*P* = 0.056); however, this numerical imbalance may reflect treatment-selection bias and the uncertainty arising from the small sample size rather than a true difference in therapeutic effect. Glucocorticoids were administered to 12.9% and 19.2% of patients in the doxycycline and azithromycin groups, respectively (*P* = 0.718). Continuous renal replacement therapy was used only in the doxycycline group (6.5%).

In terms of clinical outcomes, the 28-day hospital discharge rate was high in both groups (96.8% vs. 100%, *P* = 1.000). One patient (3.2%) in the doxycycline group died during hospitalization, while no deaths occurred in the azithromycin group (*P* = 1.000). The median length of hospital stay was 6 days in both groups (*P* = 0.691).

## 4 Discussion

This retrospective comparative study evaluated the clinical efficacy of doxycycline-based versus azithromycin-based antibiotic regimens in hospitalized patients with CPP. Baseline laboratory and imaging characteristics were comparable between the two groups. Notably, both groups presented similarly elevated C-reactive protein levels, indicating a balanced baseline systemic inflammatory status and validating the reliability of subsequent intergroup outcome comparisons. Radiologically, thoracic CT scans predominantly manifested as pulmonary consolidation accompanied by ground-glass opacities, which represented the typical imaging pattern of psittacosis. Moreover, no drug-specific radiological differences were observed between the two groups. No statistically significant differences were observed between treatment arms in key short-term outcome measures, including 28-day discharge rates, duration of hospitalization, and time to defervescence.

This finding aligned with recent cohort evidence indicating that clinical outcomes may not differ substantially across antibiotic classes in atypical pneumonia. For instance, in a multicenter retrospective cohort of 45 patients with severe psittacosis complicated by acute hypoxic respiratory failure, no significant differences in mortality or hospitalization duration were observed between patients receiving fluoroquinolones alone versus those switched to or combined with tetracyclines (Tang et al., 2023). Similarly, a recent multicenter study of 24 patients with severe *C. psittaci* pneumonia reported comparable antibiotic distribution between severe and non-severe groups, with all patients achieving favorable outcomes regardless of the specific regimen used (Xu et al., 2025). Notably, a 2025 multicenter matched cohort study by Odeyemi et al. in hospitalized community-acquired pneumonia patients demonstrated that azithromycin combined with beta-lactams was associated with lower in-hospital and 90-day mortality compared with doxycycline-based regimens (Odeyemi et al., 2026). While that study focused on broader CAP populations rather than confirmed psittacosis, it underscores the potential viability of macrolide-based therapy in intracellular pathogen pneumonia. Collectively, these observations, together with our findings, suggest that azithromycin may be a viable alternative in the short-term management of psittacosis, particularly when tetracyclines are contraindicated or unavailable.

Why azithromycin is not universally recommended as first-line therapy for CPP warrants further consideration. This may reflect historical treatment patterns, the extensive clinical experience with doxycycline, limited comparative efficacy data specifically in psittacosis, and concerns about macrolide resistance in other intracellular pathogens, rather than documented azithromycin treatment failures in CPP. The present findings should not be interpreted as evidence that azithromycin is superior or equivalent to doxycycline, but rather that it may serve as a viable alternative when doxycycline is contraindicated or unavailable. Further prospective studies are needed to clarify the optimal positioning of azithromycin in CPP treatment guidelines.

Notably, the most clinically meaningful finding was that all cases requiring invasive mechanical ventilation, ICU admission, and continuous renal replacement therapy, as well as the single in-hospital death, were confined exclusively to the doxycycline group. Although the between-group difference did not reach statistical significance, this concentrated safety signal warrants cautious and nuanced interpretation. This clustering of severe adverse clinical events is unlikely to imply inferior therapeutic efficacy or safety of doxycycline; instead, it is most plausibly attributable to substantial confounding by indication. In routine clinical practice, doxycycline serves as the primary guideline-recommended anchor regimen for patients with suspected or confirmed severe psittacosis presenting with rapid clinical deterioration. Clinicians tend to preferentially administer this first-line agent to the most critically ill patients, thereby inherently skewing baseline disease severity toward the doxycycline group (Yang et al., 2021; Yung and Grayson, 1988). Such unmeasured severity imbalance could not be fully adjusted by conventional baseline laboratory and clinical parameters in the present analysis.

Doxycycline and azithromycin have different antibacterial mechanisms, but both are widely used in the treatment of respiratory tract infections and zoonotic infectious diseases. The lack of statistically significant differences in measured outcomes observed in this study may be related to the overlap in their antibacterial spectra, and both drugs have favorable tissue penetration and bioavailability, allowing them to reach effective therapeutic concentrations in the respiratory tract and other target organs (Dominic et al., 2025). In addition, both antibiotics may also exert anti-inflammatory effects beyond their antibacterial activity, including suppression of inflammatory mediator release and attenuation of excessive systemic inflammation (Min and Jang, 2012; Singh et al., 2021). These pleiotropic effects may have contributed to the similar clinical outcomes observed in this study.

## 5 Limitations

This study has several limitations that should be acknowledged. First, the sample size of both groups was relatively small, which may lead to insufficient statistical power to detect potential small differences between the two interventions. The numerical imbalance in ICU admission rate (*P* = 0.056) may reflect true underlying differences or residual confounding, and cannot be definitively interpreted given the limited sample size. Second, the study was a retrospective analysis without randomization, which may introduce potential selection bias, although the baseline characteristics of the two groups were well balanced. Third, the study did not collect data on adverse drug reactions, so the long-term safety and tolerability of the two drugs could not be compared. Future studies should address these limitations by conducting large-scale, prospective, randomized controlled trials (RCTs) to further verify the clinical efficacy and safety of doxycycline and azithromycin.

## 6 Conclusion

Patients receiving doxycycline and azithromycin exhibited comparable baseline characteristics on measured parameters. No statistically significant differences were observed in measured short-term clinical outcomes between the two treatment groups. However, the findings should be interpreted cautiously because of limited statistical power and probable confounding by indication, particularly the preferential use of doxycycline in more severely ill patients. Both antibiotics may represent therapeutic options associated with favorable short-term outcomes. These results may guide antibiotic selection for this disease, particularly in resource-limited settings or in the absence of antimicrobial susceptibility data.

## Data Availability

The original contributions presented in the study are included in the article/supplementary material, further inquiries can be directed to the corresponding author.

## References

[B1] Beeckman D. S. Vanrompay D. C. (2009). Zoonotic Chlamydophila psittaci infections from a clinical perspective. Clin. Microbiology Infection 15 (1), 11–17. 10.1111/j.1469-0691.2008.02669.x 19220335

[B3] Chu J. Yarrarapu S. N. S. Vaqar S. Durrani M. I. (2026). “Psittacosis. StatPearls. Treasure Island (FL) ineligible companies,” in StatPearls Publishing Copyright © 2026 (Treasure Island, FL: StatPearls Publishing).

[B4] Dominic M. Srivastava R. Shah K. Naik S. M. Rout K. Ray B. (2025). Azithromycin in the management of upper respiratory tract infections (URTIs): indian real-life experience. Infect. Drug Resistance 18, 523–531. 10.2147/IDR.S488479 39898351 PMC11784350

[B5] Hammers-Berggren S. Granath F. Julander I. Kalin M. (1991). Erythromycin for treatment of ornithosis. Scand. Journal Infectious Diseases 23 (2), 159–162. 10.3109/00365549109023394 1853163

[B6] Hogerwerf L. Baan B. Van der Hoek W. (2017). Chlamydia psittaci (psittacosis) as a cause of community-acquired pneumonia: a systematic review and meta-analysis. Epidemiol. Infection 145 (15), 3096–3105. 10.1017/S0950268817002060 PMC9148753 28946931

[B7] Homma T. Yamaguchi T. Komatsu N. Hashimoto S. Doki Y. Senda K. (2011). A case of acute psittacosis with severe abdominal pain. J. Medical Microbiology 60 (Pt 4), 547–549. 10.1099/jmm.0.021154-0 21212142

[B8] Huang M. Wang Y. Lu Y. Qu W. Zou Q. Zhang D. (2025). Clinical characteristics and predicting disease severity in Chlamydia psittaci infection based on metagenomic next-generation sequencing. Infect. Drug Resistance 18, 1171–1181. 10.2147/IDR.S509879 40027914 PMC11872090

[B9] Ma K. Lu M. Li H. Yuan X. Zhang Y. Ni Q. (2025). Incidence and influencing factors of tooth discoloration in children using doxycycline: a meta-analysis. Front. Pediatrics 13, 1644231. 10.3389/fped.2025.1644231 40918671 PMC12411547

[B10] Min J. Y. Jang Y. J. (2012). Macrolide therapy in respiratory viral infections. Mediat. Inflammation 2012, 649570. 10.1155/2012/649570 22719178 PMC3375106

[B11] Odeyemi Y. E. Tekin A. Schanz C. C. Schreier D. J. Cole K. Gajic O. (2026). Comparative effectiveness of Azithromycin Versus doxycycline in hospitalized patients with community-acquired pneumonia treated with beta-lactams: a multicenter matched cohort study. Clin. Infectious Diseases 82 (4), 639–647. 10.1093/cid/ciaf252 40378193 PMC13284779

[B12] Sandman Z. Iqbal O. A. (2024). “Azithromycin. StatPearls. Treasure Island (FL) ineligible companies,” in StatPearls Publishing Copyright © 2024 (Treasure Island, FL: StatPearls Publishing).

[B13] Shapiro D. S. Kenney S. C. Johnson M. Davis C. H. Knight S. T. Wyrick P. B. (1992). Brief report: chlamydia psittaci endocarditis diagnosed by blood culture. N. Engl. Journal Medicine 326 (18), 1192–1195. 10.1056/NEJM199204303261805 1557094

[B14] Singh S. Khanna D. Kalra S. (2021). Minocycline and doxycycline: more than antibiotics. Curr. Molecular Pharmacology 14 (6), 1046–1065. 10.2174/1874467214666210210122628 33568043

[B15] Song L. Liang Q. Chen Y. Wu L. Zhou J. Wei Z. (2025). Clinical characterization and diagnosis of 14 patients with Chlamydia psittaci pneumonia. Diagnostic Microbiology Infectious Disease 113 (3), 116985. 10.1016/j.diagmicrobio.2025.116985 40617153

[B16] Tang X. Wang N. Liu G. Tan H. Li A. M. Gao Y. Q. (2023). Psittacosis caused severe community-acquired pneumonia accompanied by acute hypoxic respiratory failure: a multicenter retrospective cohort study from China. BMC Infectious Diseases 23 (1), 532. 10.1186/s12879-023-08283-z 37580698 PMC10426048

[B17] Tetracycline MotherToBaby Fact Sheets (1994). Brentwood (TN): Organization of Teratology Information Specialists (OTIS) Copyright by OTIS, January 2024.

[B18] Wormser G. P. Strle F. Shapiro E. D. (2019). Is doxycycline appropriate for routine treatment of young children with Erythema Migrans? Pediatr. Infectious Disease Journal 38 (11), 1113. 10.1097/INF.0000000000002453 31626045 PMC6814264

[B19] Wu X. Li Y. Zhang M. Li M. Zhang R. Lu X. (2020). Etiology of severe community-acquired pneumonia in adults based on metagenomic next-generation sequencing: a prospective multicenter study. Infect. Diseases Therapy 9 (4), 1003–1015. 10.1007/s40121-020-00353-y 33170499 PMC7652912

[B20] Xu T. Yuan Q. Wang J. Wu Z. Chen Z. Wang Z. (2025). Clinical characteristics and risk factors associated with severe community-acquired pneumonia infected by Chlamydia psittaci. Microbiol. Spectrum 13 (11), e0047725. 10.1128/spectrum.00477-25 40990897 PMC12584628

[B21] Yang F. Li J. Qi B. Zou L. Shi Z. Lei Y. (2021). Clinical symptoms and outcomes of severe pneumonia caused by Chlamydia psittaci in Southwest China. Front. Cellular Infection Microbiology 11, 727594. 10.3389/fcimb.2021.727594 35071027 PMC8770948

[B22] Yung A. P. Grayson M. L. (1988). Psittacosis--a review of 135 cases. Med. Journal Aust. 148 (5), 228–233. 10.5694/j.1326-5377.1988.tb99430.x 3343952

